# Characterizing Trends in Human Papillomavirus Vaccine Discourse on Reddit (2007-2015): An Observational Study

**DOI:** 10.2196/12480

**Published:** 2019-03-18

**Authors:** Yuki Lama, Dian Hu, Amelia Jamison, Sandra Crouse Quinn, David A Broniatowski

**Affiliations:** 1 Department of Family Science School of Public Health University of Maryland College Park, MD United States; 2 George Washington University Department of Engineering Management and Systems Engineering Washington, DC United States; 3 Maryland Center for Health Equity School of Public Health University of Maryland College Park, MD United States

**Keywords:** papillomavirus infections, prevention & control, cancer prevention, cervical cancer, HPV, vaccination, papillomavirus vaccines, immunology, administration & dosage, social media, health communication, infodemiology

## Abstract

**Background:**

Despite the introduction of the human papillomavirus (HPV) vaccination as a preventive measure in 2006 for cervical and other cancers, uptake rates remain suboptimal, resulting in preventable cancer mortality. Social media, widely used for information seeking, can influence users’ knowledge and attitudes regarding HPV vaccination. Little is known regarding attitudes related to HPV vaccination on Reddit (a popular news aggregation site and online community), particularly related to cancer risk and sexual activity. Examining HPV vaccine–related messages on Reddit may provide insight into how HPV discussions are characterized on forums online and influence decision making related to vaccination.

**Objective:**

We observed how the HPV vaccine is characterized on Reddit over time and by user gender. Specifically, this study aimed to determine (1) if Reddit messages are more related to cancer risks or sexual behavior and (2) what other HPV vaccine–related discussion topics appear on Reddit.

**Methods:**

We gathered all public Reddit comments from January 2007 to September 2015. We manually annotated 400 messages to generate keywords and identify salient themes. We then measured the similarity between each comment and lists of keywords associated with sexual behavior and cancer risk using Latent Semantic Analysis (LSA). Next, we used Latent Dirichlet Allocation (LDA) to characterize remaining topics within the Reddit data.

**Results:**

We analyzed 22,729 messages containing the strings *hpv* or *human papillomavirus* and *vaccin*. LSA findings show that HPV vaccine discussions are significantly more related to cancer compared with sexual behavior from 2008 to 2015 (*P*<.001). We did not find a significant difference between genders in discussions of cancer and sexual activity (*P*>.05). LDA analyses demonstrated that although topics related to cancer risk and sexual activity were both frequently discussed (16.1% and 14.5% of word tokens, respectively), the majority of online discussions featured other topics. The most frequently discussed topic was politics associated with the vaccine (17.2%). Other topics included HPV disease and/or immunity (13.5%), the HPV vaccine schedule (11.5%), HPV vaccine side effects (9.7%), hyperlinks to outside sources (9.1%), and the risks and benefit of HPV vaccination (8.5%).

**Conclusions:**

Reddit discourse on HPV vaccine encompasses a broad range of topics among men and women, with HPV political debates and cancer risk making up the plurality of the discussion. Our findings demonstrated that women and men both discussed HPV, highlighting that Reddit users do not perceive HPV as an issue that only pertains to women. Given the increasing use of social media as a source of health information, these results can inform the development of targeted online health communication strategies to promote HPV vaccination to young adult users of Reddit. Analyzing online discussions on Reddit can inform health communication efforts by identifying relevant, important HPV-related topics among online communities.

## Introduction

### Background

Long-lasting infections with high-risk human papillomaviruses (HPVs) can cause cancer in parts of the body where HPV infects cells, such as in the cervix, oropharynx, anus, rectum, penis, vagina, and vulva. Virtually all cervical cancers are caused by HPV. HPV vaccination acceptance is a critical public health issue as low completion rates of the vaccine series place adolescents and young adults at risk for HPV-associated infection and cancers. The multiple factors that influence vaccine acceptance have been examined on traditional mass media and social media, including the understudied platform, Reddit. This study is the first to examine HPV discourse on Reddit, which provides insight about attitudes of HPV vaccination among a unique population that may not be readily accessible in traditional data surveillance methods [1]. Our findings build upon the literature examining attitudes toward HPV vaccination online and in the media. Our goals were to assess whether discussions on Reddit reflected the competing narratives (cancer risk vs sexual promiscuity) described in the broader discourse [2-5] and, secondly, to document and describe the full range of HPV vaccine–related topics discussed on Reddit.

### Human Papillomavirus Vaccination Rates and Factors Affecting Uptake

The Advisory Committee on Immunization Practices (ACIP) at the Centers for Disease Control and Prevention (CDC) recommends routine HPV vaccination for all girls and boys aged 11 and 12 years, and catch-up immunization for teens and young adults up to age 26 years, to prevent HPV infection and HPV-associated cancers [6]. The vaccine series is safe and widely effective when taken before the onset of sexual activity and potential HPV exposure. However, national vaccination rates remain low, with only 49.5% of girls and 37.5% of boys completing the recommended series [6]. Increasing vaccine uptake is a preeminent national public health concern as demonstrated by the Healthy People 2020 objective to increase HPV vaccination series completion for adolescents aged from 13 to 15 years to 80% [7].

Factors contributing to lagging vaccination rates include parental concerns about vaccine safety [8], low confidence in adolescent vaccination [9], lack of provider recommendation [10], general lack of knowledge about the vaccine [11], and concerns about adolescent sexual behavior [12,13]. In particular, as HPV is sexually transmitted, some parents fear that vaccination will give license for adolescents to engage in early or risky sexual activity [2] (this has been refuted by prior research [2,14]). Owing to the stigma associated with sexually transmitted infections (STIs), parents and providers have faced challenges addressing the sexual nature of HPV [3].

This stigma is exacerbated by the fact that the initial recommendation of HPV vaccination was for girls only. Consequently, marketing of the HPV vaccine as primarily for cervical cancer prevention in 2006 has led to continued framing of HPV as a women’s health issue despite the fact that HPV affects both men and women [15,16]. A strong “gender bias” in vaccine uptake has been observed, as parents of male teens may feel their child is at low risk and the vaccine is unnecessary [17]. This enduring, gendered narrative has contributed to low vaccine uptake and opportunity loss for cancer prevention among men, who are increasingly affected by HPV-related anal, penile, and oropharyngeal cancers [18].

### Traditional and Social Media

The media are influential in relaying HPV-related cancer risk communication and contributing to fears of adolescent sexual behavior, including promiscuity concerns. Prior content analyses of US and Canadian newspapers have found that HPV-related articles mentioning sexual promiscuity concerns ranged from 30% to 60% [4,5,19], detracting from a focus on more pertinent details of vaccination (eg, reducing HPV-associated cancers and vaccination schedule and benefits). The media’s framing of the HPV vaccine as competing narratives of adolescent sexual behaviors and cancer risk may have implications for influencing public knowledge, awareness, and beliefs on HPV vaccination.

Although HPV vaccination has been studied on other media, social media may have a particularly strong impact on public perception of HPV vaccination risks. In total, 69% of adults use some form of social media [20], and research has shown that an increasing number of people seek health information online [21,22]. Furthermore, the open nature of social media provides a critical opportunity to investigate personal attitudes and beliefs regarding HPV vaccination. Thus, an understanding of the online discourse regarding HPV vaccination can aid public health communicators in their efforts to address public misconceptions.

Reddit is a popular online forum that allows users to post, share, and rank content through a voting system. Reddit has 542 million monthly visitors, nominating it as the fifth most visited website in the United States [23,24]. Although HPV has been studied on social media platforms such as Twitter, YouTube, and Pinterest [25-27], to our knowledge, no published study has examined Reddit content related to HPV vaccination. Reddit data capture attitudes and trends that are not readily accessible in traditional data surveillance methods, as highlighted in previous studies that used Reddit data [28,29]. Moreover, Reddit’s features allow users to post anonymously, allowing users to disclose personal behaviors more readily [28,29].

Reddit’s relative lack of restrictions on posting requirements and its significant number of publicly available posts related to HPV vaccination make it a critical site to assess HPV discourse among its user base. Reddit is typically associated with a male adolescent culture as 67% of users are men and 59% of users are between the ages of 18 and 29 years [30], providing an opportunity to communicate with a group that is eligible for vaccination, at risk of HPV-associated cancers, and otherwise hard to reach. In a survey of the Reddit community [31], 83.5% of participants identified themselves as aged between 18 and 34 years. Of these, 80.4% identified themselves as male. Thus, an analysis of Reddit provides insight into the male perspective of HPV vaccination on social media, an understudied area in the previous literature where women have been the focus.

### Study Objectives

This study was the first to utilize Reddit data to gain a better understanding of online public discussions of the HPV vaccine, including determining whether online discussions mirror previous research and media coverage. We observed how the HPV vaccine is characterized on Reddit over time and by user gender. Furthermore, to the extent that those two topics may not be representative of the total HPV-related discourse on Reddit, we conducted an additional topic analysis. Our research questions (RQs) are as follows:

RQ1: Are Reddit messages more related to cancer risks or to sexual behavior?)

RQ2: What other topics characterize the discussion on HPV vaccination on Reddit?)

Studying cancer risks, sexual activity, and other emerging topics from Reddit content would provide greater insight into extant online discussions about HPV vaccine, which can inform concerted, tailored health communication efforts to promote HPV vaccination and improve uptake rates.

## Methods

### Data Source

We used Reddit as our main data source. Unlike microblogging social media platforms such as Twitter, Reddit does not limit message length, allowing users to express their opinions in depth. Communities can be organized by different topics through “subreddits,” which are forums dedicated to a specific topic such as news (/r/news) or HPV (/r/HPV). Reddit also has a strong community-based moderation culture; repetitive messages or unrelated messages will often be removed by moderators of each subreddit. Although it is possible that messages in our sample are due to malicious actors, such as bots and trolls [32], Reddit’s moderation culture also helps to mitigate these concerns. Therefore, Reddit comments are a promising data source for understanding how a segment of the online community perceives the risks of HPV and the risks and benefits of the HPV vaccine. The study was determined to be research that is exempt from the Institutional Review Board at the George Washington University (IRB-180804).

### Data Collection

We downloaded all recent Reddit comments from January 1, 2007, to September 30, 2015, using a platform which allows researchers to collect and share complete Reddit datasets for research purposes [33]. At the time of analysis in 2016, the most up-to-date data were culled, resulting in our use of data from 2007 to 2015. From the total set of all messages, we identified a subset of HPV-related messages by filtering all comments containing the text “hpv” or “human papillomavirus”. From the set of hpv-related messages, we further identified a subset of vaccine-related messages by filtering messages for the string “vaccin.”

### Data Analysis

Our first analysis was designed to answer RQ1.

Our aim was to determine whether HPV-related messages discussed sexual behavior or cancer risk more often. First, 2 annotators (YL and AJ) manually annotated 50 messages and selected an initial set of keywords associated with both topic areas. The same annotators then annotated another 350 messages, in rounds of 50 each, adjusting the keywords and refining coding techniques. We agreed upon a final set of keywords as follows: a single keyword, “cancer,” was sufficient to identify messages pertaining to cancer. Messages pertaining to sexual behavior included keywords derived from the “purity” category in the Moral Foundation Dictionary [34]: “piety, pious, purity, pure, clean, sterile, sacred, chaste, saint, innocent, unclean, slut, whore, dirty, impiety, impious, profane, promiscuity, promiscuous, adulter, unchaste, sexual, sex, intercourse, coitus, lovemaking, and premarital.” Using these specific keywords, annotators initially began annotating for promiscuity; however, owing to a limited number of messages, this topic was expanded to include all sexual activity related to HPV (including promiscuity). Through this iterative process, we created a codebook to identify messages as either pertaining to cancer risk, sexual activity, or other (Table 1). There was moderate agreement on messages indexing sexual behaviors, Cohen kappa=0.62 (95% CI 0.54 to 0.69), and high agreement on messages indexing cancer risk, kappa=0.81 (95% CI 0.74 to 0.88).

Next, we employed Latent Semantic Analysis (LSA) [35], a commonly used technique in natural language processing, to measure the semantic similarity between each Reddit message and the keyword lists. Specifically, we rendered the set of all HPV-related Reddit messages into a term-document matrix and transformed this corpus into a “latent semantic space.” When preparing the corpus for the LSA analysis, we removed all but the 500 most frequent unigrams. We next applied term frequency-inverse document frequency weighting to the corpus. Finally, consistent with the past literature, we retained dimensions 2 to 101 when computing the Singular Value Decomposition underlying LSA. We used the underlying LSA space to measure the cosine similarity between each Reddit post and each keyword list and calculated the average similarity score per month (see Multimedia Appendix 1).

In addition, we developed a classifier to assess the gender of the user posting each message. This tool helped us understand the overall difference between male and female user discussions related to the HPV vaccine. The classifier works by identifying gender-indicating keywords and expressions included in Reddit messages. For instance, statements such as “As a man…” or “I am a straight woman…” are used as indicators of a user’s gender (Multimedia Appendix 2). In some instances, there was not enough information to identify a user’s gender with confidence, so those users were classified as having an unknown gender.

**Table 1:** Qualitative codebook and sample messages for Research Question 1.

Domain	Description	Sample messages^a^
Cancer risk	Messages are related to HPV^b^ and relationship to cancer and HPV vaccine. This includes concerns related to cancer and awareness of cancer risk. Comments can be any related type of cancer: cervical, throat, penile, and anal.	“it's not just genital warts, the hpv vaccine can prevent cervical cancer.”; “the vaccines don't prevent all causes of cervical cancer, they protect against some strains of hpv which can cause cervical cancer. also, they only started giving that vaccine to women over 25 recently.”
General sexual behavior	Messages are related to HPV and sexual activity including discussions of age of sexual debut, risky sexual behavior, multiple sex partners, and associations with promiscuity. Messages need an explicit connection to HPV. Can include screening behavior if it is related to sexual activity.	“I've only had one sexual partner in my history and we were both virgins, so i'm certain i'm clear. not that hpv is a death sentence or anything, but i'd rather not have to deal with it *.* ”

^a^Messages have been lightly edited to protect user anonymity.

^b^HPV: human papillomavirus.

We manually annotated over 100 messages to test the accuracy of the classifier. A total of 2 annotators (DH and DB) read messages identified by the classifier and determined that 43 of 50 messages were correctly classified as “male” and 45 of 50 messages were correctly classified as “female.” This indicates an overall accuracy of 88% (see Multimedia Appendix 3).

We also developed a classifier to assess the age of users into 7 age categories (10 to 18, 18 to 24, 25 to 34, 35 to 44, 45 to 54, 55 to 64, and 65 and older) consistent with Reddit surveys [31]. When reading the gender-indicating expressions (eg, “I am 27 years old”), we observed the linguistic features related to age-indicating expressions were sometimes complicated and open to misclassification. For example, some expressions discussing time or gestational age were coded as ages, resulting in the classifier achieving 67% accuracy. Nevertheless, the current accuracy is higher than the random baseline of 7 categories (14.28%; Multimedia Appendix 4). This classifier, which is being developed, cannot be used to reach a definite conclusion of the age groups. Instead, it can be applied to report Reddit users’ demographic information for the scope of this study.

Our second analysis addressed RQ2.

To further explore the topics discussed on Reddit, we used Latent Dirichlet Allocation (LDA) [36], a Bayesian topic model, to automatically segment Reddit messages into 10 probabilistic “topics” (using unigram features; hyperparameter values were alpha=.1 and η=0.01). LDA assigns each word token to one of these topics, allowing one to summarize the content of each Reddit post. After examining models with 20 and 50 topics, we determined that a 10-topic model best captured the variety of discussion subjects with minimal redundancy.

For each month, we calculated how many word tokens belonged to each topic. A total of 2 annotators (YL and AJ) read the sample messages and top word tokens of each topic to determine the theme of each LDA topic. The proportional topic weight of each year was calculated by the following equation:

proportional topic weight of Topic X=(The frequency of all words that belongs to Topic X)/(The total frequency of all words in given years)

The detailed results and interpretation are reported below.

## Results

### Research Question 1: Cancer Risk and Sexual Activity

**Figure 1:**
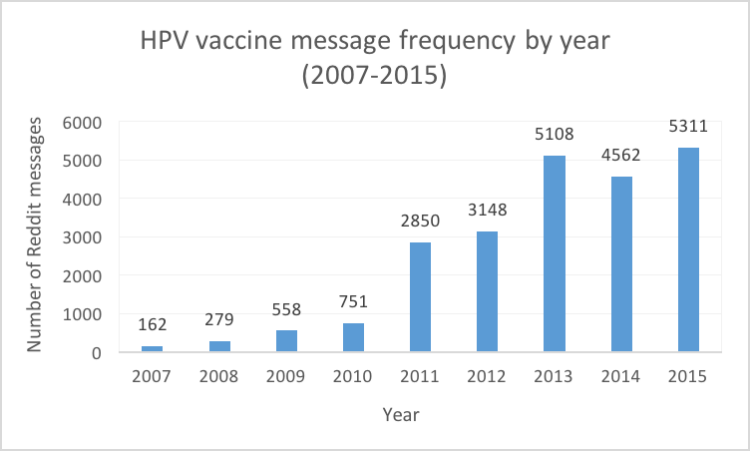
Number of Human papillomavirus (HPV) vaccine messages by year.

Our final dataset included 22,729 messages filtered to include “hpv” or “human papillomavirus” and “vaccin”. Mitigating concerns about behavior by malicious actors, such as bots, we found that no two messages in our dataset are exactly the same, a common indicator of bot behavior [37]. The number of messages varied by year and increased over time, from 162 messages in 2007 to a peak of 5311 messages in 2015 (Figure 1). Among those posting HPV vaccine messages (n=12,952), 53.0% (6869/12,952) were male, 8.8% (1143/12,952) were female, and 38.1% (4940/12,952) were of unknown gender (Table 2). Nearly 17% of users were aged from 25 to 34 years, followed by 11.2% of users aged 18 to 24 years, although 60.0% of users did not specify their age (Table 2).

In manual annotation, it was clear that messages describing cancer risk were more identifiable than messages discussing HPV-related sexual behavior. Cancer risk messages tended to focus on specific themes, including the relationship between HPV infection and cancer incidence (especially related to specific HPV strains), and on HPV vaccination as a resource to prevent future cancer incidence. Messages about sexual behaviors were more varied. Initially, we attempted to code for instances strictly related to HPV vaccine and sexual promiscuity but found very few instances (<5 in the first 100 messages) that we were unable to do so. The widespread use of sarcasm and irony on Reddit also made it difficult to know the meaning of some messages out of context. Many of the messages we encountered incorporated the moral foundation keywords, but only to refute the idea that HPV vaccine promotes promiscuity. To capture the complexity of this topic, we broadened our coding scheme to include any discussion of sexual activity and HPV vaccination and were able to identify a wider range of subjects including safe sex practices, personal stories, and debates over sexual risk.

**Table 2:** Age and gender distribution of the Reddit sample data.

Age group (years)	Female users	Male users	Unspecified gender	Age subtotal
10 to 18	128	339	57	524
18 to 24	293	862	291	1446
25 to 34	376	1489	297	2162
35 to 44	103	549	65	717
45 to 54	42	195	26	263
55 to 64	7	37	2	46
65 and above	7	13	0	20
Unspecified age	187	3385	4202	7774
Gender subtotal	1143	6869	4940	12,952

**Figure 2:**
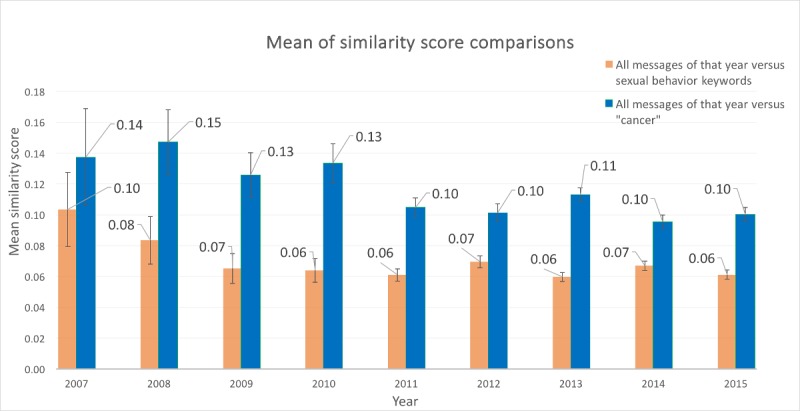
Comparison of message similarity between human papillomavirus (HPV) cancer risk and sexual activity on Reddit by Latent Semantic Analysis (LSA).

The LSA analysis showed that cancer is significantly more discussed than sexual activity (Table 3). Discussions about sexuality and cancer did not typically co-occur: The cosine similarity score between the cancer risk and sexual activity keyword lists in the semantic space was −0.01, indicating that the 2 lists were essentially orthogonal. Discussions about HPV vaccine were more similar to the keyword “cancer” than to the sexual purity keywords, particularly after 2007 (Figure 2). However, the average monthly Reddit message discussed neither cancer nor sexuality (cosine similarity values were 0.11 and 0.08, respectively). These low similarity scores indicate that other topics must exist in the corpus that are neither about cancer nor sexual behavior. Furthermore, we observed a decrease in average similarity to both cancer and sexuality over time, indicating that other topics of discourse may have emerged within these forums.

In general, we did not notice a significant difference between genders in discussions of cancer and sexual activity (all *P* values were greater than .05, except in 2012; note that we would expect one *P* value to be less than .05 owing to chance alone; Figure 2).

**Table 3:** *t* test of similarity of messages between human papillomavirus (HPV) cancer risk and sexual activity on Reddit by Latent Semantic Analysis (LSA).

Year	Message count	Sexual similarity, mean	Cancer similarity, mean	Yearly *t* test score
2007	162	0.10	0.14	−1.70
2008	279	0.08	0.15	−4.83^a^
2009	558	0.07	0.13	−6.84^a^
2010	751	0.06	0.13	−9.26^a^
2011	2850	0.06	0.11	−11.97^a^
2012	3148	0.07	0.10	−9.35^a^
2013	5108	0.06	0.11	−19.20^a^
2014	4562	0.07	0.10	−10.25^a^
2015	5311	0.06	0.10	−14.64^a^

^a^*P*<.001.

### Research Question 2: Selected Topic Analysis

In our close reading of messages for manual annotation, we recognized that although messages included keywords related to cancer risk or sexual activity, there were additional topics that extended beyond the scope of either topic (Table 4). Some of these conversations were narrowly focused on HPV vaccination, for instance, debates over whether vaccinating the entire population was a cost-effective prevention method or discussions of vaccine efficacy related to particular strains of HPV. Other conversations were focused on sexual activity and health, in general. We observed a subset of conversations that detailed personal experiences with STIs and described the various steps individuals had taken to avoid all types of STIs (eg, condom use, HPV vaccination, and regular STI testing). Another major topic was related to circumcision, as some users felt that primary prevention measures such as HPV vaccination reduced the need to circumcise (as circumcision is often cited as a method to reduce STI infection). Finally, another subset of conversations was focused on broader political and philosophical discussions that sometimes used HPV vaccination as a talking point. For instance, political discussions of the role of government sometimes questioned vaccine mandates as infringing on parental rights as part of a larger justification for libertarianism. Alternatively, users would discuss sexual education, access to contraceptives, and HPV vaccination as talking points in discussions of abortion rights (Table 4). Although these conversations were not specifically focused on HPV vaccination, we believed they reflect the broader discourse on the topic and were an important area of study.

**Table 4:** Summary of human papillomavirus (HPV) vaccine topics generated by the Latent Dirichlet Allocation (LDA) approach.

Topic name	Topic keywords	Description
General vaccine debate (Topic 0)	peopl, immun, diseas, get, like, flu, one, hpv, caus, children	Broadly concerned with vaccine decision making; Vaccine safety; Risks of side effects; Importance of vaccination; Personal stories
HPV^a^-related cancer and genital warts (Topic 1)	hpv, strain, cancer, caus, wart, protect, type, infect, genit, genit-wart	HPV strains related to cancer and genital warts; Strains covered by vaccine
Sharing government links (Topic 2)	http, www, http-www, gov, cdc, cdc-gov, nih, www-cdc, nih-gov, nlm	Vaccine concerns corroborated by links to CDC^b^ and PubMed research articles
HPV cancer prevention (Topic 3)	cancer, cervic, cervic-cancer, hpv, get, women, prevent, caus, hpv-vaccin, men	Gender-related vaccine and HPV concerns; cervical cancer; other HPV related cancers; recommendations for men and women
HPV-specific vaccine debate (Topic 4)	effect, gardasil, studi, report, gt, hpv, death, year, side, hpv-vaccin	Adverse events linked to HPV vaccination; Side effect risk; Vaccine efficacy; Debating facts (fewer personal stories than topic 0).
HPV vaccine political debate (Topic 5)	peopl, hpv, make, gt, would, like, hpv-vaccin, think, one thing	Parental vaccine rights; Reach of federal power; Abortion debate; Sexual education policy; General political debate; Individual politicians; Corruption
Sharing general links on HPV (Topic 6)	http, com, www, http-www, org, amp, en, hpv, comment, wikipedia	HPV concerns with links to news stories; Links to other Reddit threads; Generally sharing single links rather than a list of links (Topic 2)
HPV vaccination schedule (Topic 7)	get, hpv, hpv-vaccin, year, got, doctor, go, shot, age, girl	Age of vaccination; Preventive screenings; Medical advice; Personal stories; Deciding whether to vaccinate
Circumcision debate (Topic 8)	circumcis, men, gt, risk, women, male, hiv, infect, hpv, benefit	Risks and benefits of circumcision; Likelihood of contracting STI^c^; Cancer prevention
Sexual behaviors including HPV (Topic 9)	get, hpv, sex, test, condom, peopl, like, know, partner, std	Discussions of sexual behaviors; STI prevention; Vaccination; Condom use; Relationship norms

^a^HPV: human papillomavirus.

^b^CDC: Centers for Disease Control and Prevention.

^c^STI: sexually transmitted infection.

In addition to the diversity in topics, it appears users were turning to Reddit for a variety of reasons: some were posing questions and were looking for answers; others seemed to want to debate; some just wanted to discuss topical issues in the news; and still others seemed to want to make jokes. Messages ranged from one-word responses to posts a few thousand words long. Some were based on personal experiences and anecdotes, and others were based on facts and statistics, often linking to cited research. The understanding gained from reading HPV vaccine–related messages led us to our second research question, as we realized we needed to explore the full range of topics that were being discussed on Reddit.

**Figure 3:**
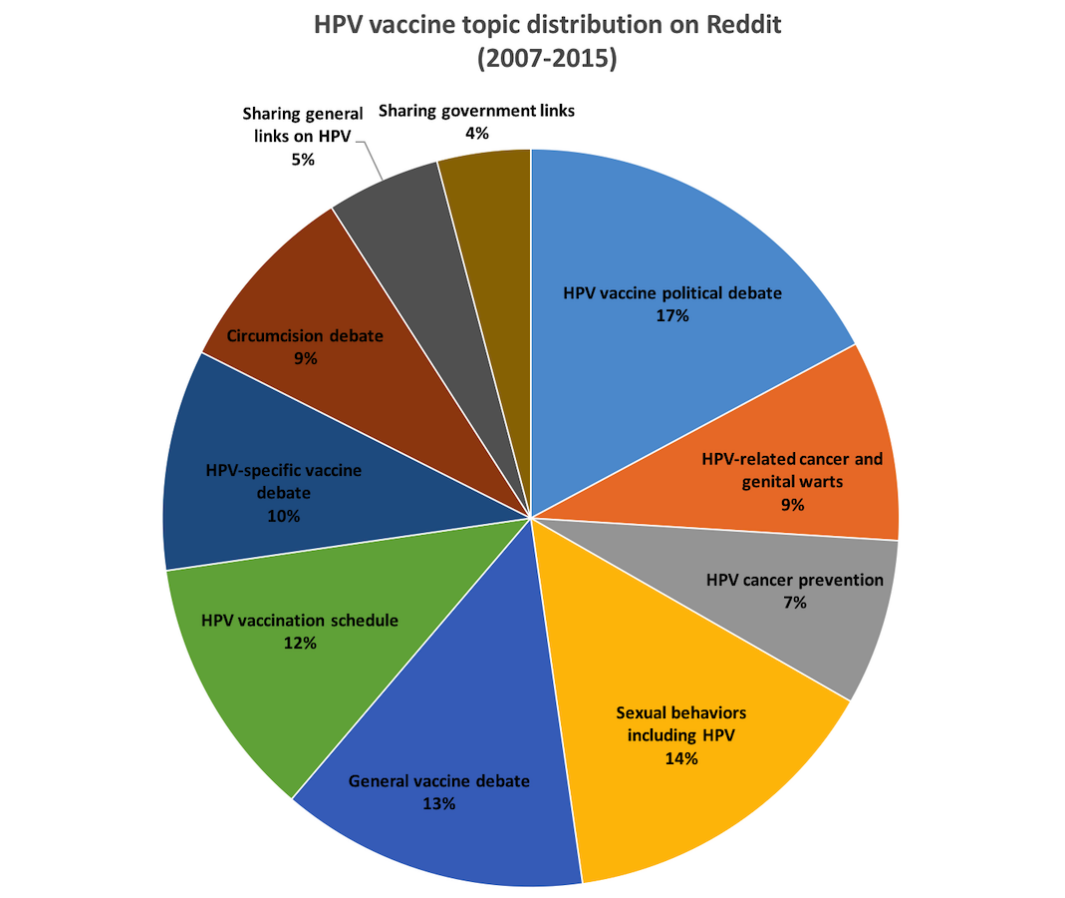
Human papillomavirus (HPV) vaccine topic distribution on Reddit.

Expanding upon the LSA findings, the LDA topics (Table 4) generated a more comprehensive list of all HPV vaccine–related topics of interest. The most widely discussed topic was HPV-related political debate (17.2%), wherein users posted messages on political topics including vaccine policy, abortion rights, and sex education policy. Cancer was the second most widely discussed (16.1%), after combining the two closely related topics of HPV strains causing cancer and genital warts and HPV cancer prevention. This was followed by sexual activity and preventive behaviors (14.5%), which focused on behaviors such as using condoms, receiving HPV vaccination, and regular testing and screenings for STIs. The fourth most popular topic was HPV disease and immunity (13.5%), which included broad concerns with diseases and infections associated with HPV. The fifth was the vaccine schedule related to HPV (11.5%), which included messages on age of vaccination initiation and requesting information on vaccination from physicians. The sixth, vaccine side effects and risks (9.7%) included messages on adverse events linked to HPV vaccination. The seventh topic was related to messages that included use of government or research (eg, CDC and PubMed) papers and general websites (eg, Wikipedia and news sites) to corroborate user opinions (9.1%). The final topic discussed circumcision, particularly the risks and benefits as related to STI and cancer prevention (8.5%) (Figure 3).

The political debate surrounding HPV vaccines peaked in 2011 (24%) and decreased in 2013 (14%), when the number of messages plateaued (Multimedia Appendix 5). General HPV vaccine messages peaked in 2008 (20%), whereas general vaccine messages sharply decreased during 2008 (8%) and increased in 2009 (22%). Discussion of sexual behavior sharply increased in 2010 (18%) and 2014 (17%). Circumcision debate sharply increased in 2012 (11%; Multimedia Appendix 5).

Across the LDA topics, notable spikes were apparent in certain time periods (ie, 2008, 2009, 2011, 2013, and 2014). We observed peaks for certain years across LDA topics (Multimedia Appendix 5). Specifically, we observed an increase in the discussion related to HPV-associated cancers and genital warts in 2008 (10%; Multimedia Appendix 5). Furthermore, increases were observed across HPV cancer (11% and 0.092) and sexual activity discussions (20% and 18%, respectively) in 2010 (Multimedia Appendix 5). Discussions of cancer increased in 2013 (8% and 10%, respectively) and 2014 (17%).

## Discussion

### Principal Findings

Our findings emphasize increased discussion of HPV-related cancer risks on Reddit, in contrast with the mainstream media’s focus on sexual behavior linked to HPV vaccine. Of the total set of HPV vaccine–related Reddit messages, 16.1% were focused on cancer risk topics and 14.5% were focused on HPV related to sexual activity. However, even when combined, these two topics represented less than one-third of the total HPV vaccine–related messages on Reddit. LSA findings did not show significant gender differences in discussions of HPV-related sexual behaviors and cancer risks, though discussions of both topics decreased over time. We identified additional topics that provide insight into the broader HPV discourse on Reddit, which included HPV disease and immunity, vaccine schedule, side effects, risks and benefits of vaccination, and the circumcision debate (Figure 3), providing further public health surveillance of nuanced HPV attitudes on Reddit.

Our LSA findings demonstrated that, on Reddit, cancer is significantly more discussed than sexual activity, consistent with the majority of prior research [14,38,39]. Although HPV cancer risk prevention and HPV-related sexuality are presented as competing narratives in the broader media discourse, these two topics alone do not reflect the focus of the majority of HPV-related conversations on Reddit. Indeed, the political debate regarding vaccination was the most discussed topic on Reddit. The HPV vaccine has been the subject of political discussion since its introduction in 2006 [40]. Messages spiked in 2008 for the HPV vaccine debate, perhaps associated with media attention surrounding state consideration for school vaccination mandates for middle-school age girls [41]. The following year also saw spikes in messages for general vaccine debate, perhaps associated with the introduction of a second HPV vaccine, Cervarix, in 2009. That same year, Gardasil was approved for boys aged 9 to 26 years to reduce genital warts; however, the ACIP did not recommend routine vaccination among males until 2011 [40]. We observed continued increases in discussion through 2011 and 2012, most likely due to CDC HPV vaccine recommendations for boys in fall 2011 coinciding with the 2012 presidential campaign, which incited debates around vaccination [40]. Increases in cancer discussions during 2013 and sexual activity in 2014 do not coincide with major policy changes or announcements and thus warrant additional research on factors that contributed to these trends.

Our analyses did not support the idea of HPV as primarily a woman’s health issue on Reddit, highlighting the differences between narratives on Reddit and general public discourse. This discrepancy is surprising, given the initial recommendation and marketing of the vaccine toward girls to prevent cervical cancer [15] and given the majority of Reddit users are adolescent males. Furthermore, until recently, media coverage of HPV vaccine has largely been focused on women, particularly regarding promiscuity [42] even with expanded vaccine recommendations to include males. Our LDA findings also challenge the notion that HPV is perceived as an issue affecting women exclusively, with sizable discussion on Reddit on HPV prevention for men related to circumcision. The circumcision debate indicates broader concerns of men concerned with STI and cancer prevention related to HPV. These results further emphasize the difference between discussions on Reddit and media representations on the HPV vaccine.

The implications of inconsistencies between HPV vaccination discussion on Reddit compared with media outlets are significant. Continued media coverage of controversies may detract from focus on benefits of vaccination, which may translate to lower rates of vaccine uptake and increased incidence of cancer [43]. Public health agencies can utilize traditional and digital media to share targeted and tailored health messages promoting HPV vaccination to the broader population. Our findings demonstrated a wide spectrum of viewpoints, ranging from evidence-based posts to subjective personal experience posts with varying accuracy. Users reported questions about HPV prevention strategies (eg, vaccination vs using condoms), best practices for regular screenings (eg, Pap tests), and the benefits of vaccination while considering potential side effects. By highlighting the topics most salient to Reddit users, public health communication efforts can be targeted to suit the needs of this online community. In addition, the range and extent of messages from users seeking information highlights a gap in accessible credible information online. These messages on Reddit can pose a challenge for a user to navigate and make an informed decision regarding vaccination.

Critical to vaccine uptake is the receipt of health care provider (HCP) recommendation [42], which may increase awareness of the benefits of HPV vaccine as well as cancer risks of nonvaccination. Despite recommendations being a key driver in uptake, physicians are more likely to recommend other vaccines (ie, tetanus and diphtheria) compared with HPV vaccine [44]. Lacking HCP recommendations or accurate knowledge, users may turn to online forums such as Reddit seeking advice, which may be filled with inaccurate information. In addition, without provider and public health engagement in these forums, misconceptions surrounding HPV vaccination may proliferate, fueling vaccine hesitancy.

Our analysis of Reddit posts can inform health communication efforts tailored to users’ queries. Topics such as HPV vaccination as cancer prevention can be promoted whereas other topics, such as fear of vaccine side effects, can be combated with accurate messaging. Our findings suggested an increasing interest in HPV vaccination on Reddit over time, indicating a need for public health communication through social media platforms such as Reddit. Public health officials can develop health communication strategies that engage users, such as answering questions through Ask Me Anything events on Reddit or enlisting experts or well-known public figures to promote vaccination. Providers can share accurate HPV information and engage with patients regarding their questions and concerns regarding HPV vaccination, particularly through national campaigns and events. Massey et al found that HCPs were less likely to utilize hashtags and engage in Twitter chat events, resulting in decreased online presence and missed opportunities to productively engage with users [45].

Given the increasing number of people using social media and seeking health information online, public health practitioners and agencies need to take advantage of these opportunities to connect with users online to advocate for accurate, timely information. This may help to combat misconceptions related to HPV vaccination behavior. For example, in posts related to the vaccine schedule, users questioned the need for age cutoffs for HPV vaccines, a question which can be addressed with clear messaging about eligibility, especially given the recent Food and Drug Administration approval for expanded use of individuals aged from 27 to 45 years [46]. The continued failure to address these communication needs may contribute to increased vaccine hesitancy, further decreasing vaccination rates and, subsequently, increasing HPV-related morbidities. Although the increased effort of targeted engagement with users online will require more resources (ie, dedicated time) from public health agencies, the opportunities to reach and impact HPV vaccination greatly outweigh the challenges.

### Limitations

The messages are drawn from Reddit users and do not necessarily represent the range of attitudes and beliefs across the broader population. Consistent with Barthel et al, our dataset revealed that the majority of users who posted HPV vaccine messages were males (53%) compared with females (8.8%) [30]. However, the fact that Reddit users skew younger in age, with 59% users between the ages of 18 and 29 years, is in line with target audiences for vaccination, particularly at the catch-up stage (aged 18 to 26 years) [30]. Our findings do not examine other demographic variables besides binary gender (ie, male or female) and only examined messages from 2007 to 2015. During this period, we noticed spikes in conversation that did not coincide with major policy recommendations or noteworthy events, warranting a further examination of underlying causes of these upticks in discussion.

Future studies would benefit by examining other demographic variables across further time points. Although our data limited us to examine only gender and age, future research can be expanded to parse messages by user characteristics such as age and geographical area to examine complex HPV attitudes with another level of granularity. This is especially important given the disparities in HPV vaccine uptake in rural areas compared with urban areas. Furthermore, sentiment analysis may provide insight regarding differences in message content (positive, neutral, and negative) across users. Topic analysis can also be applied across social media to examine any differences in discussion across different platforms.

### Conclusions

This study provided critical insight about HPV vaccination discourse on Reddit over time. In particular, we found Reddit users were discussing a wider variety of topics, beyond cancer risks and sexual activity, in contrast to prevailing media focus. Reddit can be a surveillance tool to examine emerging trends among users. In response, health communication stakeholders can utilize this platform to address concerns of Web-based communities to the extent that they are representative of the broader public, instead of concerns of the news cycle. Finally, our results indicate that HPV is discussed among both men and women on Reddit, and it is heartening that HPV is not perceived as an issue that only pertains to women. These findings may inform the development of strategies to address HPV vaccine information, while dispelling misinformation and misconceptions, to increase vaccination and promote sexual health online. As young adults increasingly use Web-based resources to seek health information, public health communication efforts are needed to leverage this opportunity to deliver timely, accurate health information to internet communities.

## Appendix

Multimedia Appendix 1 Latent Semantic Analysis (LSA) similarity results.

Multimedia Appendix 2 Gender classifier accuracy assessment.

Multimedia Appendix 3 Development strategy of the gender classifier.

Multimedia Appendix 4 Annotation to assess accuracy age classifier.

Multimedia Appendix 5 Latent Dirichlet allocation (LDA) results.

## Abbreviations

ACIP: Advisory Committee on Immunization Practices
CDC: Centers for Disease Control and Prevention
HCP: health care provider
HPV: human papillomavirus
LDA: Latent Dirichlet Allocation
LSA: Latent Semantic Analysis
NIH: National Institutes of Health
STI: sexually transmitted infection
