# Supplementary material for: Characterizing Trends in Human Papillomavirus Vaccine Discourse on Reddit (2007-2015): An Observational Study
Source: JMIR Public Health Surveill. 2019 Mar 18;5(1):e12480. doi: 10.2196/12480 (PMC6533775; doi:10.2196/12480)
Supplement: Multimedia Appendix 3 [file publichealth_v5i1e12480_app3.docx]

Supplement File 4: **development strategy of the gender classifier** (Step 1: Reconstruct the Reddit data based on the username of the messages. Step 2: For each user, we use linguistic-explicit self-reporting regular expressions to scan through all messages contributed from that user to identify certain demographic traits like age, gender or race. For example, if one user has used the expression once like “I’m 25.”, “I am a woman,” “my husband” The algorithm would record the expression and give the user a temporal evaluation of “25”, “Female.” To ensure maximum precision of the method, we would only consider message with self-reporting grammar, e.g., “I am,” “I’m” and “my”. During this step, each regular expression is tested and modified to filter out the most common noise. Step 3: After going through all messages of all users, we calculate the overall evaluation of each user. Because of our strict restriction in regular expressions. Some users would remain **unknown** after step 2 and step 3.)
